# Effects of visually induced motor imagery-based brain-computer interface training on motor function in patients with incomplete spinal cord injury: a small-sample exploratory trial

**DOI:** 10.3389/fneur.2026.1700249

**Published:** 2026-02-03

**Authors:** Yuyang Zhao, Chong Sun, Yunfeng Bi, Yongxiang Zhang

**Affiliations:** 1 Department of Rehabilitation Medicine, The Affiliated Hospital of Qingdao University, Qingdao, Shandong, China; 2 Department of Spinal Surgery, The Affiliated Hospital of Qingdao University, Qingdao, Shandong, China

**Keywords:** brain-computer interface, EEG, incomplete spinal cord injury, motor imagery, neurological recovery

## Abstract

**Objective:**

This study aimed to investigate the effects of visually induced motor imagery (MI)-based brain-computer interface (BCI) training on the neurological recovery of patients with incomplete spinal cord injury (iSCI), and to preliminarily explore the underlying neural mechanisms.

**Methods:**

A single-center, single-blind, small-sample exploratory trial was conducted, enrolling 11 patients with iSCI who were randomly assigned to either the experimental or control group. The experimental group received visually induced BCI training based on a MI paradigm, while the control group received visually guided MI training combined with passive lower limb movements. Both groups underwent interventions five times per week for 4 weeks. Clinical assessments, including the American Spinal Injury Association (ASIA) motor/sensory scores, Berg Balance Scale (BBS), and Functional Ambulation Category (FAC), were conducted before and after the intervention. Simultaneously, electroencephalography (EEG) data were collected to analyze brain engagement, functional connectivity, and time-frequency characteristics, aiming to elucidate the neuromodulatory effects of BCI training.

**Results:**

After the intervention, both groups showed significant improvements in brain engagement, with the experimental group demonstrating greater enhancement. Compared with before rehabilitation training, the levels of θ waves in both groups significantly increased after rehabilitation training, while the levels of β waves significantly decreased (*p* < 0.05), especially in areas related to exercise planning and sensory integration. The connections between brain regions in the delta and theta frequency bands were significantly enhanced, and the density of brain network connections was significantly increased (*p* < 0.05) particularly in regions associated with motor planning and sensory integration. Clinically, all functional scores improved significantly in both groups (*p* < 0.05), and the experimental group showed superior improvement in ASIA motor and sensory scores, BBS, and FAC levels compared to the control group (*p* < 0.05).

**Conclusion:**

Visually induced MI-based BCI training effectively promotes neurological recovery in patients with iSCI, as evidenced by enhanced brain network reorganization, modulation of cortical excitability, and activation of motor-related neural rhythms. This study confirms the feasibility and safety of this intervention strategy and offers a novel direction for iSCI rehabilitation.

**Clinical Trial Registration:**

Chinese Clinical Trial Registry (ChiCTR), identifier: ChiCTR2400095010.

## 1 Introduction

Spinal cord injury (SCI) is a severe central nervous system impairment that often leads to significant deficits in motor, sensory, and autonomic functions (1). Compared with complete injuries, iSCI preserves partial neural conduction pathways, offering a certain potential for spontaneous recovery (2). However, despite some degree of neurological improvement, conventional rehabilitation strategies remain insufficient to fully activate the residual neural circuits and achieve meaningful functional restoration (3, 4). Therefore, it is imperative to explore targeted interventions capable of enhancing neuroplasticity and promoting neural network reorganization, in order to improve quality of life and functional independence in iSCI patients.

The recovery of motor function in iSCI patients mainly depends on spinal cord plasticity (such as axonal regeneration and synaptic reorganization) and brain functional reorganization (such as the remodeling of the motor cortex representation area). The typical rehabilitation showed its limitations of iSCI. The limitations included that traditional rehabilitation training (such as exercise therapy and physical therapy) has limited effects on patients in the chronic stage, and about 50% of patients have moderate to severe motor disabilities left; it relies on “passive movement” or “assisted movement,” making it difficult to activate the brain network above the injury level; it lacks active regulation of the neural plasticity mechanism and cannot specifically enhance the excitability of key brain regions.

In recent years, BCI technology has emerged as a promising neuromodulation tool in neurorehabilitation (5). By recording neurophysiological signals such as EEG and translating them into control commands for external devices, BCI enables direct communication between the brain and the external environment (5, 6). For individuals with iSCI, BCI offers not only the potential to bypass damaged descending pathways but also the ability to reactivate cortical function and facilitate the reorganization of sensorimotor circuits (7, 8). Previous studies have shown that BCI paradigms incorporating multimodal feedback-particularly visual and proprioceptive-can enhance cortical activation, improve functional connectivity within motor-related networks, and ultimately promote motor recovery (9, 10). Nonetheless, the precise neuromodulatory mechanisms underlying BCI training in iSCI remain unclear, and the individualized optimization of intervention parameters-such as feedback modality, training frequency, and task design-continues to be a major focus of ongoing research (11).

This single-center, single-blind, small-sample exploratory trial aims to evaluate the effects of multimodal BCI training-combining visual feedback and proprioceptive stimulation-on motor and neurological function in individuals with iSCI, while exploring the potential underlying mechanisms. The intervention group received a MI-based BCI training protocol with real-time EEG monitoring and lower-limb proprioceptive stimulation, whereas the control group received visually guided MI training combined with passive limb movement. The neural plasticity mechanisms of MI-BCI include: activity-dependent plasticity and multimodal integration effects. Through clinical assessments and EEG-based network analyses, this study seeks to elucidate how BCI interventions may facilitate cortical reorganization, enhance cortical excitability, and improve functional connectivity. The findings aim to provide empirical evidence for the clinical feasibility and mechanistic basis of BCI in iSCI rehabilitation and contribute to the development of personalized, precision rehabilitation strategies in the future.

## 2 Materials and methods

### 2.1 Inclusion and exclusion criteria

The inclusion criteria for this study were: patients who met the 2011 diagnostic criteria of the ASIA and were classified as ASIA grades B-C, with a confirmed diagnosis of cervical, thoracic, or lumbar SCI based on computed tomography or magnetic resonance imaging. Patients were required to be in the recovery phase, with stable vital signs and Kinesthetic and Visual Imagery Questionnaire score ≥ 55 after the acute phase, and aged between 18 and 70 years, and willing to voluntarily participate in the study with informed consent from both the patients and their families.

Exclusion criteria included the presence of severe complications such as respiratory failure, heart failure, acute myocardial infarction, renal failure, severe pulmonary infections, or malignancies that significantly affect quality of life. Also excluded were patients with lower limb spasticity scores above 2 according to the modified Ashworth scale, those with limited lower limb joint mobility due to trauma, patients with cognitive dysfunction (Mini-Mental State Examination score ≤ 22), those with intracranial metal implants, or those in the spinal shock phase.

This study was a single-center, single-blind, small-sample exploratory trial. A total of 12 patients with traumatic iSCI who were admitted to the Department of Rehabilitation Medicine at the Affiliated Hospital of Qingdao University and voluntarily participated in this study between January 1, 2025, and April 1, 2025, were enrolled. All participants were in the recovery phase of SCI (3 months−1 year post-injury). A block randomization method was used for grouping, with the random sequence generated by MATLAB software. Participants were assigned to the experimental and control groups in sequential order, with 6 participants in each group. The grouping and intervention process were carried out by clinical researchers, and outcome assessors were blinded to the group allocation. During the study, one participant in the control group withdrew voluntarily due to discharge, resulting in a total of 11 participants completing the study. The demographic characteristics of the two groups are detailed in Table 1. No significant differences were found between the groups in terms of gender, age, ASIA classification, or MI function scores (*p* > 0.05), as shown in Table 2.

**Table 1:** Demographic characteristics of patients with spinal cord injury.

**ID**	**Control group**					**Experimental group**				
	**Sex**	**Age**	**Level of injury**	**KIVQ**	**ASIA classification**	**Sex**	**Age**	**Level of injury**	**KIVQ**	**ASIA classification**
1	Male	37	T10	85	B	Male	37	T10	84	B
2	Male	62	L3	84	C	Male	70	C4	86	C
3	Male	66	T12	86	C	Male	52	T10	89	B
4	Female	52	T10	82	B	Male	57	C4	83	B
5	Male	55	C5	87	B	Male	24	T11	85	B
6						Male	66	T12	83	C

**Table 2:** Statistical comparison of demographic characteristics between control and experimental groups.

**Group**	***n***	**Sex (male/female)/*n***	**Age (years)**	**KIVQ**	**ASIA grade**	**Motor imagery ability score**
Control group	5	4/1	51.50 ± 17.67	84.8 ± 1.92	0.33 ± 0.52	47.20 ± 3.92
Experimental group	6	6/0	54.4 ± 11.19	85.0 ± 2.28	0.40 ± 0.55	46.83 ± 6.94
*p*-value		0.2967	0.7590	0.880	0.8402	0.4372

### 2.2 Ethical approval

This study was approved by the Ethics Committee of the Affiliated Hospital of Qingdao University (Ethical Approval No.: QYFYEC2024-55) and registered with the Chinese Clinical Trial Registry (ChiCTR; Registration No.: ChiCTR2400095010). All research activities were conducted in strict accordance with national regulations, including the “Ethical Review Measures for Biomedical Research Involving Humans” (2016) and other relevant laws. Prior to enrollment, all participants were fully informed by the researchers about the study's purpose, procedures, potential risks, and possible benefits, and they voluntarily signed written informed consent forms. For the use of images, audio, and video materials in the study, participants also signed a separate consent form for the use of such information, ensuring that their personal privacy and portrait rights were fully protected in compliance with legal requirements.

### 2.3 Training protocol

Participants were assigned to either the conventional motor rehabilitation + MI training group (control group) or the conventional motor rehabilitation + BCI training group (experimental group) according to the pre-designed grouping scheme. Both groups underwent a 4-week rehabilitation training program, with five training sessions per week, each session consisting of various training activities. Each participant completed a total of 20 treatment sessions. All participants received conventional motor rehabilitation training, including regular muscle strength training, positional transfer training, standing training with assistive devices, and robot-assisted walking training (Motomed, Xi'an ZhenTec Intelligent Technology Co., Ltd.). Each conventional training session lasted 30 min and was conducted once daily under the guidance of experienced rehabilitation therapists.

The control group received 15 min of MI training in addition to the conventional training. During the training, participants wore an EEG cap and in-ear headphones while sitting on the Motomed device. In a passive state, they engaged in cycling-like training. Participants were guided to imagine alternating movements of their lower limbs on the pedals without actively exerting force. The Motomed device was set to passive mode with a constant speed to enhance somatosensory feedback.

**Figure 1:**
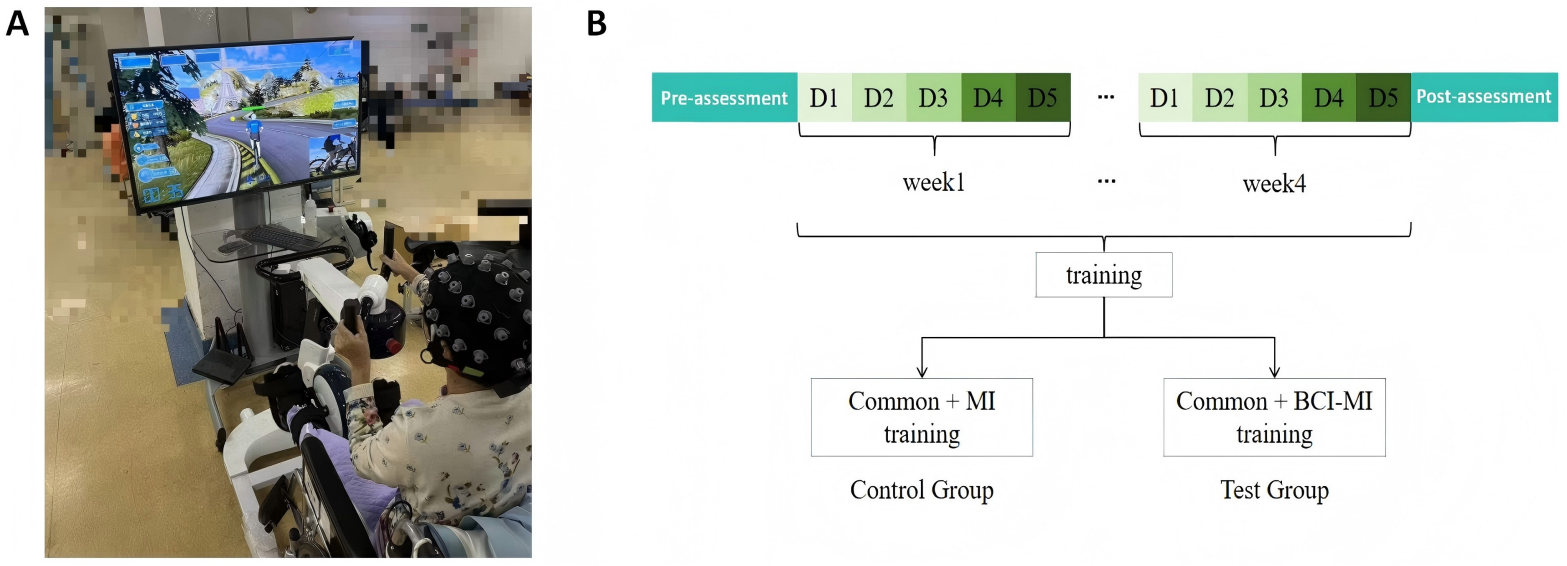
**(A)** Visual and EEG-Guided BCI cycling training setup based on motor imagery. The setup illustrates the brain-computer interface cycling training paradigm, where participants wear an EEG cap and headphones. Under auditory cues, they focus on the screen while engaging in a cycling simulation game. Without active physical effort, participants activate brain regions through motor imagery of bilateral lower limb pedaling. Real-time EEG signals are collected and fed back to the Motomed device, which adjusts the cycling speed in proportion to brain signal engagement. The speed of the on-screen avatar corresponds to the strength of the brain signal until the endpoint is reached. **(B)** Data collection phases: pre-assessment, training, and post-assessment.

The experimental group received 15 min of BCI training in addition to the conventional rehabilitation. The ZhenTec-R1 BCI upper and lower limb rehabilitation training system (Xi'an ZhenTec Intelligent Technology Co., Ltd.) was used for the training (12). Participants wore an EEG cap and in-ear headphones and, in response to auditory cues, focused on a screen displaying an interactive cycling game. By imagining lower limb pedaling movements without actively exerting force, participants activated brain activity. Real-time EEG signals were collected and fed back to the Motomed device, with the device's speed proportional to the brain signal engagement. Simultaneously, the speed of the character in the game was synchronized with the intensity of the brain signal, continuing until the character reached the finish line (Figure 1A). To ensure consistency in rehabilitation training duration and intensity, both the control and experimental groups completed 45 min of rehabilitation training daily, including conventional training and additional MI or BCI training.

### 2.4 Data collection

In this study, data collection was conducted in three phases: baseline assessment, rehabilitation training period, and post-assessment (Figure 1B). The baseline assessment was performed the day before the rehabilitation training, and the post-assessment was scheduled on the first day after the completion of the rehabilitation training. During both the baseline and post-assessments, clinical functional evaluations were carried out for both groups, including the ASIA motor score, ASIA sensory score, BBS, and FAC score. Additionally, resting-state EEG data were collected during both the baseline and post-assessments with a sampling rate of 500 Hz. EEG data collected during the rehabilitation training period were task-related data with a sampling rate of 250 Hz. During task-based data collection, participants wore a 30-channel EEG cap for data recording. The EEG signals were acquired using an amplifier and transmitted to a computer for analysis. Subsequently, the data underwent preprocessing and feature extraction using specialized software. The clinical evaluation was completed by two resident physicians in the rehabilitation department, who were unaware of the patient grouping. First, the two resident physicians in the rehabilitation department received clinical evaluation training to ensure consistency in evaluation methods such as the ASIA motor score, ASIA sensory score, MBI scale score, BBS scale score, and FAC scale grading. After the rehabilitation training, the two resident physicians in the rehabilitation department conducted relevant clinical evaluations on the patients simultaneously. If the scores were similar, the average was taken. If the difference was large, the chief physician who was proficient in the scoring procedure re–evaluated the patients.

### 2.5 Data preprocessing

Data preprocessing was conducted using Matlab 2021a and the EEGlab toolbox. First, the electrode coordinate file was loaded and matched with the electrode positions of the cap. Next, the ERPLAB filter was applied for notch filtering to remove 50 Hz power line interference. A 6th-order Butterworth bandpass filter was then applied with a frequency range of 1–45 Hz to retain the main frequency bands of brain activity. Eye movement artifact electrodes (HEOL, HEOR) were excluded to remove irrelevant electrodes. Independent Component Analysis was used to remove independent components related to artifacts. During preprocessing, the signals were also referenced to the average of all electrodes to reduce common noise across electrodes. Subsequently, the data was segmented into 2-s epochs, and each epoch was checked for amplitude. Any epoch with an absolute amplitude exceeding 75 μV was discarded.

**Figure 2:**
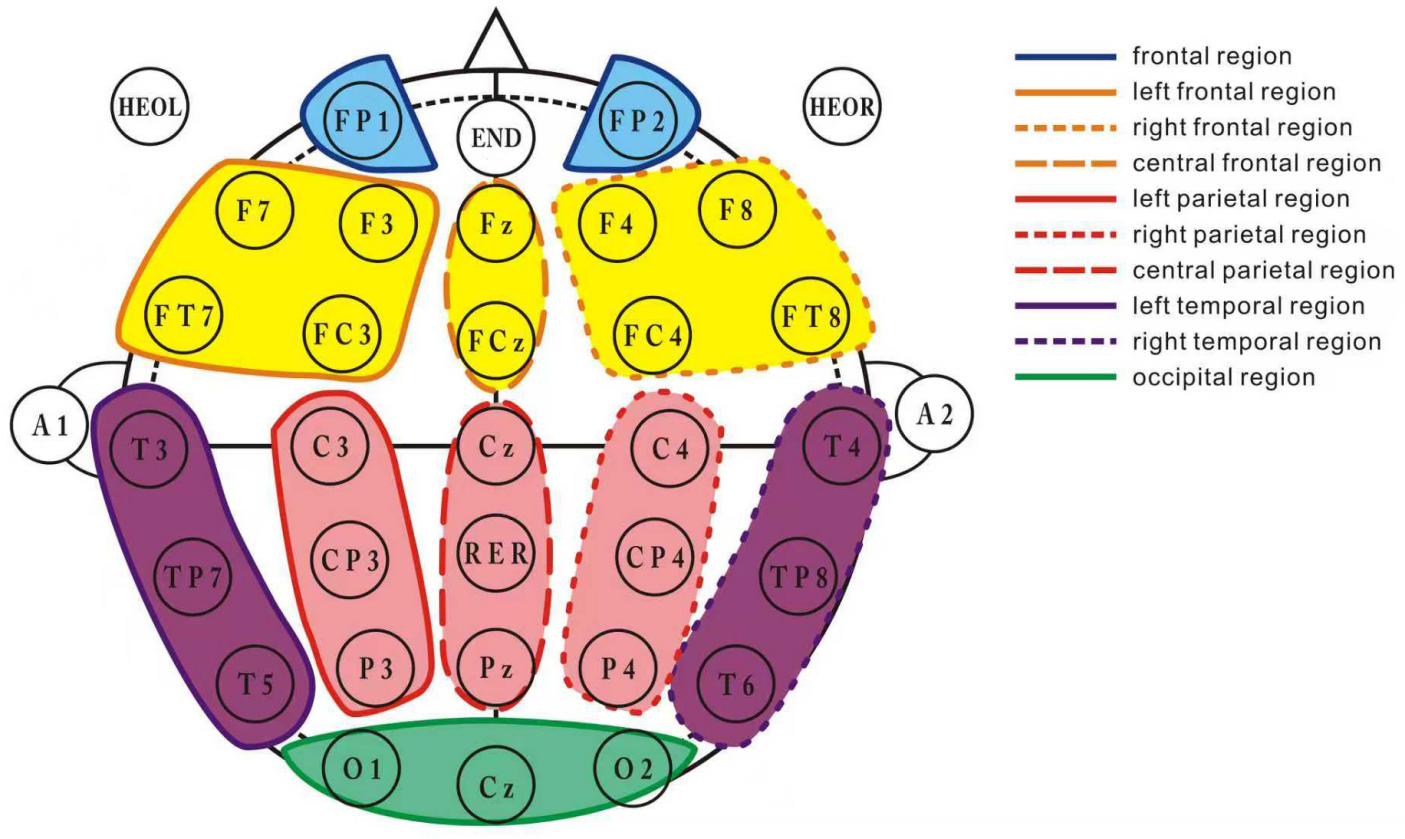
Brain region division layout. This figure illustrates the division of the 30-channel EEG signals into 10 distinct brain regions. The channels are grouped as follows: frontal region (Fp1, Fp2), left frontal region (F3, F7, FC3, FT7), right frontal region (F4, F8, FC4, FT8), central frontal region (Fz, FCz), left parietal region (C3, CP3, P3), right parietal region (C4, CP4, P4), central parietal region (Cz, CPz, Pz), occipital region (Oz, O1, O2), left temporal region (T3, T5, FT7), and right temporal region (T4, T6, FT8). Each brain region's corresponding EEG channels are marked to facilitate the understanding of the electrode configuration for data collection.

The EEG signals were divided into the following frequency bands: delta (1–4 Hz), theta (4–8 Hz), alpha (8–15 Hz), beta (15–30 Hz), and gamma (30–45 Hz) (13). After preprocessing, the 30-channel EEG signals were divided into 10 brain regions according to the following distribution (Figure 2): prefrontal region (Fp1, Fp2), left frontal region (F3, F7, FC3, FT7), right frontal region (F4, F8, FC4, FT8), central frontal region (Fz, FCz), left parietal region (C3, CP3, P3), right parietal region (C4, CP4, P4), central parietal region (Cz, CPz, Pz), occipital region (Oz, O1, O2), left temporal region (T3, T5, FT7), and right temporal region (T4, T6, FT8).

### 2.6 Data analysis

MI is a cognitive processing process in which participants subjectively imagine performing a movement without actually engaging in physical motion or muscle contraction. Based on the way it is represented, MI can be categorized into two types: muscle movement perception imagery and visual MI (14). This study analyzes EEG data from both resting state and task state to comprehensively evaluate changes in neural activity and levels of active participation before and after rehabilitation training (15).

In the resting state analysis, EEG data were used to assess changes in therapeutic effects before and after the rehabilitation intervention. The resting state EEG analysis primarily included the following feature extractions: (1) Power Spectral Density of EEG, calculating power changes in five frequency bands: delta (1–4 Hz), theta (4–8 Hz), alpha (8–15 Hz), beta (15–30 Hz), and gamma (30–45 Hz), to reflect the neural activity levels in different frequency ranges; (2) Quantitative EEG metrics, including theta/alpha ratio [TAR = Power (theta)/Power (alpha)] and theta/beta ratio [TBR = Power (theta)/Power (beta)], to characterize the trend of neural activation state changes; (3) Functional connectivity analysis based on resting state EEG, further assessing the network topology between brain regions; (4) the global efficiency is a commonly used indicator in brain network connectivity topology diagrams. The d_ij_ is the shortest path length between node i and node j, and the formula for global efficiency is as follows:

${\text{E}}_{\text{glob}}=\frac{1}{N\left(N-1\right)}\;\underset{i\neq j\in G}{\sum }\;\frac{1}{d_{ij}}$. In this study, the weighted Phase Lag Index (wPLI) was used to calculate brain network connectivity strength. The wPLI reflects the consistency of phase differences between different brain regions, with values ranging from 0 to 1. A higher wPLI value indicates more stable and synchronous connections. The calculation formula is as follows: $\text{wPLI}=\frac{\left|\langle ℑ\;\left(X\right)\rangle \right|}{\langle \left|ℑ\;\left(X\right)\right|\rangle }=\frac{\left|\langle |ℑ\;\left(X\right)|sign\left(ℑ\;\left(X\right)\right)\rangle \right|}{\langle \left|ℑ\;\left(X\right)\right|\rangle }$, where ℑ (*X*)represents the imaginary component of the cross-spectrum between regions *x*_(*t*)_ and *y*_(*t* )_.

In the task state analysis, EEG data were primarily used to assess the level of focus and active MI ability during the training process, reflecting the level of brain engagement during the intervention. The brain engagement refers to the attention index, and previous studies have shown that human attention is correlated with the Alpha (7–13 Hz) and Beta (14–30 Hz) frequency bands. The attention intensity feature can be calculated by the energy ratio of Alpha and Beta frequency signals (17). This metric dynamically reflects whether participants are actively engaged in the task scenario and helps identify the degree of cooperation during rehabilitation training and the activation of potential neural plasticity mechanisms.

## 3 Statistical analysis

Statistical analyses were performed using MATLAB 2020a software. The distribution characteristics of continuous data were assessed using the Shapiro-Wilk normality test. If the data followed a normal distribution, they were expressed as mean ± standard deviation (Mean ± SD). Before performing inter-group comparisons, Levene's test was conducted to confirm the homogeneity of variances. If the conditions were met, repeated-measures analysis of variance was used to test the interaction and main effects, and the Bonferroni method was used for *post-hoc* tests. For within-group comparisons, the normality of change scores (or difference scores) was tested. If the data followed a normal distribution, the paired *t*-test was applied. For data that did not follow a normal distribution, they were described as median (interquartile range) [M (QL, QH)]. The Mann-Whitney *U* test was used for between-group comparisons, and the Wilcoxon signed-rank test was used for within-group comparisons. Categorical data were presented as frequencies (n), and the chi-square test was used for between-group comparisons. All tests were two-sided, and the significance level was set at α = 0.05. Specifically, the sex, age, KIVQ, ASIA grade, and Motor Imagery Ability Score before training was compared between two group using independent samples *t*-test. Additionally, after training, the BBS, FAC score, ASIA motor score, ASIA sensory score, Global efficiency and Brain engagement were also compared using independent samples *t*-test.

## 4 Results

### 4.1 Functional scores

Before treatment, there were no significant differences between the two groups in ASIA motor index scores, ASIA sensory index scores, BBS scores, and FAC walking function levels (*p* > 0.05). After 4 weeks of treatment, all scores, except for the BBS score in the control group, showed significant improvement in both groups (*p* < 0.05). The experimental group demonstrated superior improvement in all scores compared to the control group (*p* < 0.05). The detailed results are shown in Tables 3–5.

**Table 3:** ASIA motor and sensory index score.

**Group**	**Pre-treatment**	**4 weeks post-treatment**	***p*-value**
**Motor**			
Control group	52.40 ± 25.04	57.60 ± 20.45	0.0544
Experimental group	52.67 ± 22.11	65.17 ± 28.20	0.0437
*p*-value	0.1128	0.0359	
**Sensory**			
Control group	179.20 ± 39.02	184.20 ± 35.19	0.0476
Experimental group	139.50 ± 56.99	168.67 ± 69.07	0.0355
*p*-value	0.2257	0.0255	

**Table 4:** Berg balance scale score.

**Group**	**Pre-treatment**	**4 weeks post-treatment**	***p*-value**
Control group	5.80 ± 4.49	8.20 ± 3.54	0.0511
Experimental group	5.00 ± 2.77	10.17 ± 5.76	0.0402
*p*-value	0.2207	0.0435	

**Table 5:** FAC walking function classification.

**Group**	**Pre-treatment**	**4 weeks post-treatment**	***p*-value**
Control group	0.20 ± 0.40	0.80 ± 0.75	0.0458
Experimental group	0.23 ± 0.47	1.17 ± 1.07	0.0416
*p*-value	0.1577	0.0369	

### 4.2 Resting-state EEG data analysis

In the resting-state EEG analysis, we selected 10 brain regions and calculated and compared the relative power changes of Delta, Theta, Alpha, Beta, and Gamma frequency bands to assess the differences in neural activity between the two groups before and after rehabilitation training.

**Figure 3:**
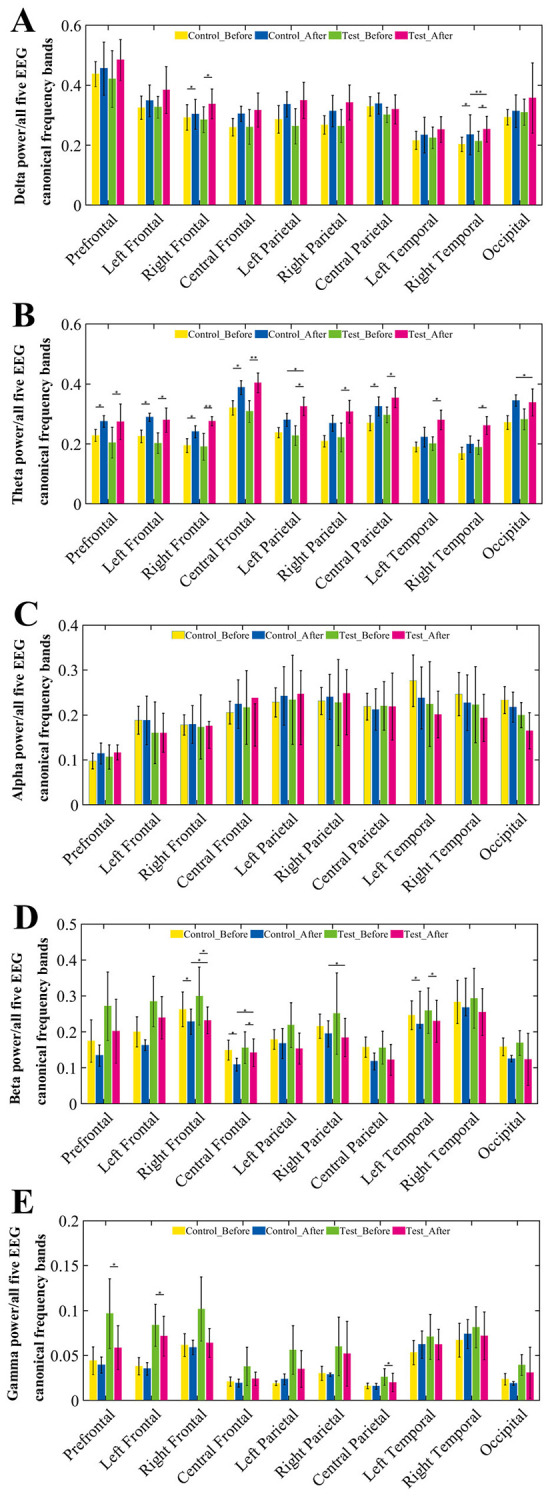
Relative power changes across EEG frequency bands in 10 brain regions before and after training. **(A–E)** Group comparisons of relative EEG power across 10 brain regions in five canonical frequency bands: **(A)** Delta (1–4 Hz), **(B)** Theta (4–8 Hz), **(C)** Alpha (8–15 Hz), **(D)** Beta (15–30 Hz), and **(E)** Gamma (30–45 Hz). For each frequency band, the relative power was calculated under resting-state conditions at pre-training and post-training phases for both control and BCI groups. Each subplot illustrates the mean relative power with error bars across four conditions: control group (pre, post) and BCI group (pre, post).

As shown in Figure 3, compared to pre-training, Theta waves exhibited a trend of increased relative power across all brain regions in both groups. Further statistical analysis revealed that the experimental group showed significant changes in Theta wave power across all brain regions (*p* < 0.05), with particularly significant changes in the right frontal and central frontal regions (*p* < 0.01). However, the control group did not show significant differences in the left and right parietal and temporal regions, with statistical significance observed in the other brain regions (*p* < 0.05). Additionally, after training, the relative power of Beta waves decreased across all brain regions in both groups, particularly in the right frontal, central frontal, and left temporal regions (*p* < 0.05), predominantly in the M1 motor area of the brain.

**Figure 4:**
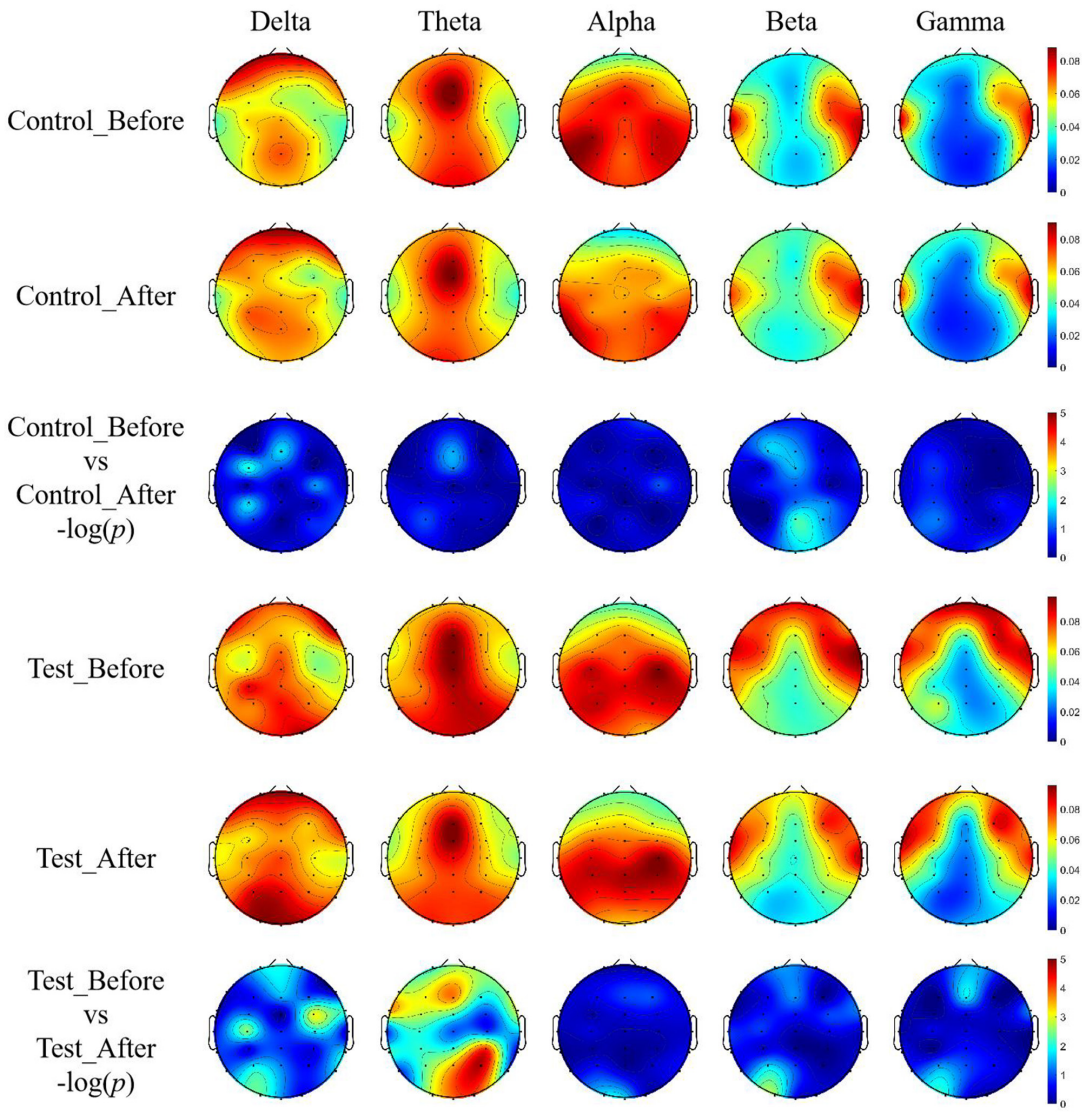
EEG topographic maps of relative power across five frequency bands with unit of DB. Topographic distributions of EEG relative power in delta (1–4 Hz), theta (4–8 Hz), alpha (8–15 Hz), beta (15–30 Hz), and gamma (30–45 Hz) bands are shown for both groups before and after training.

The topographic map of EEG (Figure 4) further demonstrates significant differences in the whole-brain power distribution before and after training in the experimental group in the Theta frequency band, with statistical results showing –log (*p*) > 2.9999, confirming the reliability of the bar chart analysis in Figure 3.

**Figure 5:**
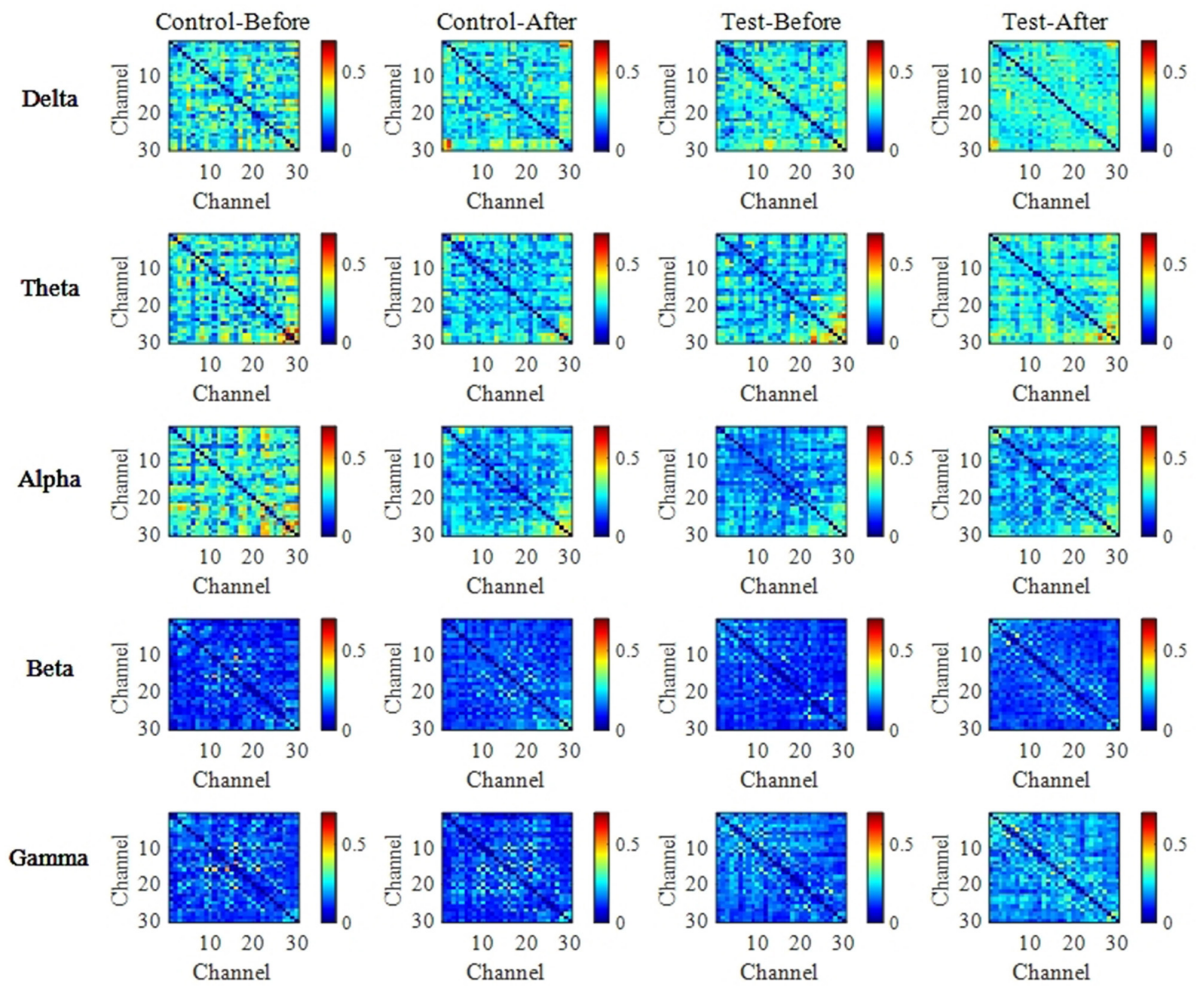
Functional connectivity matrices based on wPLI before and after training. Weighted phase lag index (wPLI) was calculated for each frequency band (Delta, Theta, Alpha, Beta, Gamma) using wavelet decomposition. Connectivity matrices illustrate the functional brain network patterns of the control and BCI groups before and after training. The color intensity in the matrix represents the strength of phase synchronization between brain regions, with red indicating stronger connectivity and blue indicating weaker connectivity.

After wavelet decomposition to extract signals from each frequency band (Delta, Theta, Alpha, Beta, and Gamma), the wPLI was calculated for each sample, and significance tests were performed. This allowed for the construction of the functional connectivity network's connectivity matrix and topological structure maps. As shown in Figure 5, the color intensity in the functional connectivity matrix reflects the strength of the connections between brain regions, with red indicating enhanced connections and blue indicating weakened connections.

The results show that before rehabilitation training, both groups exhibited strong brain region connections, with the experimental group showing a significantly higher number of strong connections compared to the control group. Pre-training, the experimental group's functional connectivity was mainly characterized by overall weak connectivity, with most cells in the connectivity matrix appearing lighter in color, indicating low phase synchronization between brain regions. However, some regions still showed locally enhanced connectivity, suggesting that some communication capabilities between brain regions remained despite the neural injury, although the overall network showed poor global integration, which may impact complex cognitive and motor functions.

After rehabilitation training, the experimental group showed increased network connectivity in all frequency bands, with more cells in the connectivity matrix appearing darker, indicating significantly enhanced interactions between brain regions. At the same time, the network structure exhibited better global integration, with more brain regions involved in synchronized activities, likely reflecting brain plasticity changes induced by the rehabilitation training. Furthermore, the functional connectivity map before training exhibited notable left-right asymmetry, which improved after training, suggesting that this change may be closely related to the recovery of motor function.

**Figure 6:**
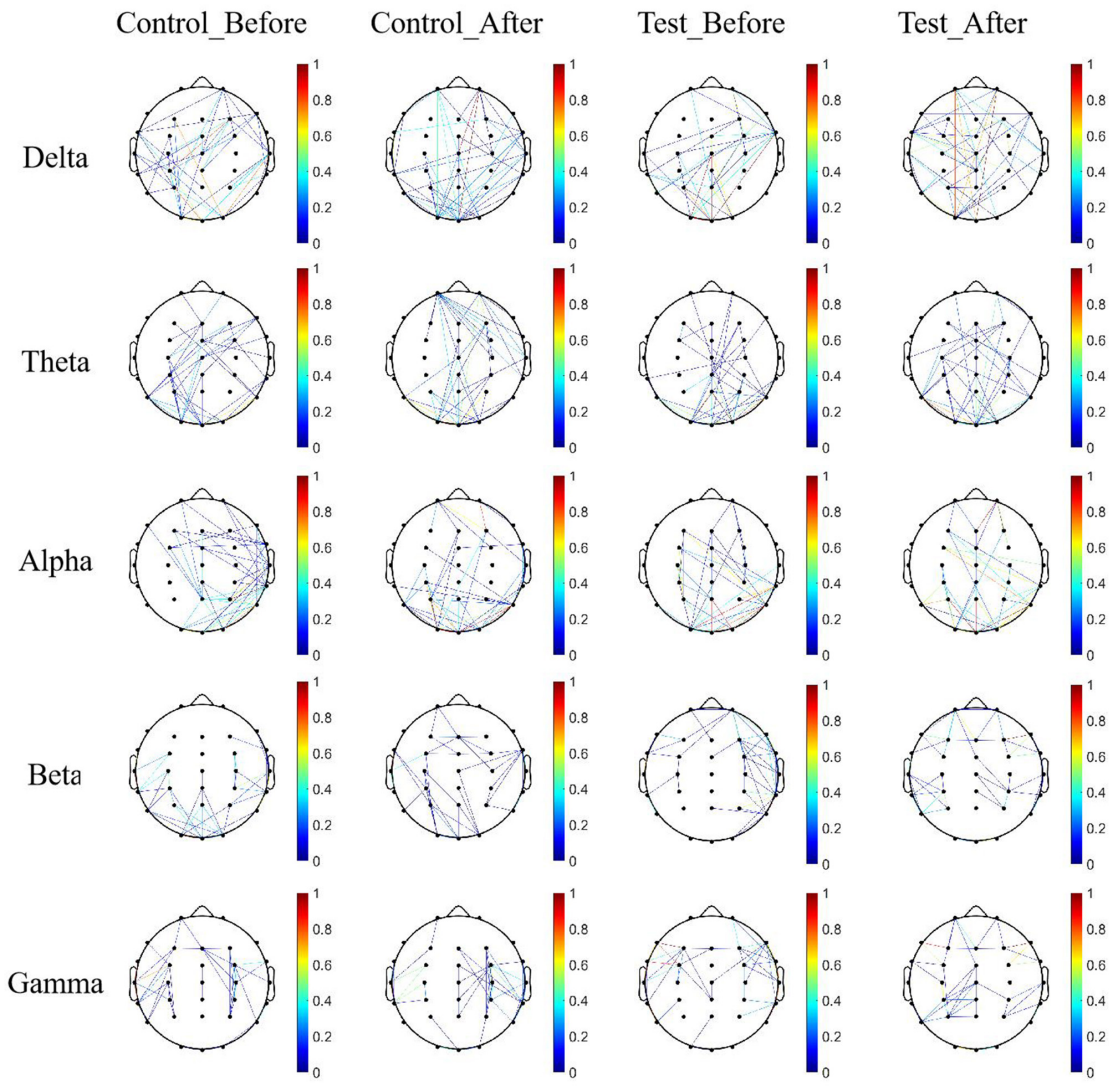
Brain network connectivity maps across five EEG frequency bands before and after training. Functional brain network graphs were constructed based on wPLI-derived connectivity matrices using the Bsmart toolbox. To emphasize significant connections, a sparsity threshold was applied to each participant's matrix by setting connections below a defined threshold to zero, while retaining higher values as weighted links. The resulting sparse matrices were aggregated and further thresholded using the threshold_proportional function from the Brain Connectivity Toolbox (BCT), with the sparsity threshold set at 0.3 to ensure sufficient intra- and inter-group connectivity.

To more intuitively visualize the brain network's connectivity characteristics, brain network diagrams corresponding to the above connectivity matrices were generated using the Bsmart toolbox. A threshold was set according to the sparsity of connections, and any connections in the connectivity matrix of each subject below this threshold were set to 0, with the remaining connection values preserved as network weights. The sparse matrices obtained from each subject were then summed, and the connectivity matrix was filtered using the threshold_proportional function from the Brain Connectivity Toolbox. To ensure that both groups had good network connectivity effects within and between the groups, a network sparsity threshold of 0.3 was set, and brain network connection maps were generated, as shown in Figure 6.

From Figure 6, it can be observed that in the Delta and Theta frequency bands, the connectivity density of brain networks in both groups increased after treatment, particularly within the parietal brain region. Additionally, connections between the parietal region and other brain regions also increased, and the symmetry of the network significantly improved compared to pre-treatment, with the experimental group showing stronger network connectivity.

In the Alpha, Beta, and Gamma frequency bands, brain network connections were mainly concentrated in the limbic regions of the brain. After 4 weeks of rehabilitation training, the connected regions gradually shifted toward the central parietal region, with a more pronounced shift in the experimental group compared to the control group, particularly in the Alpha frequency band.

Quantitative analysis of the topological indices of the brain networks was performed, followed by statistical comparison, and the results are detailed in Table 6. From the global efficiency of the networks, the experimental group showed significantly more pronounced differences before and after treatment.

**Table 6:** Global efficiency of brain network.

**Group**	**Delta**	**Theta**	**Alpha**	**Beta**	**Gamma**
Control group_Pre	0.46 ± 0.22	0.41 ± 0.21	0.53 ± 0.24	0.45 ± 0.11	0.40 ± 0.13
Control group_Post	0.55 ± 0.26	0.47 ± 0.15	0.45 ± 0.18	0.32 ± 0.15	0.34 ± 0.11
*p*-value	0.0425	0.0413	0.0530	0.0521	0.0544
Experimental group_Pre	0.47 ± 0.31	0.40 ± 0.23	0.52 ± 0.15	0.44 ± 0.21	0.39 ± 0.16
Experimental group_Post	0.58 ± 0.18	0.51 ± 0.17	0.41 ± 0.26	0.30 ± 0.20	0.33 ± 0.18
*p*-value	0.0326	0.0321	0.0476	0.0509	0.0516

### 4.3 Task-state EEG data analysis

The BCI rehabilitation training system based on MI drives the lower limb rehabilitation robot to perform lower limb circular movements through changes in the brain involvement index. Therefore, this study conducted a pre- and post-comparison analysis of the changes in the brain involvement index for both groups of patients during the training process (five sessions per week, for 4 weeks, totaling 20 training sessions), with the results shown in Supplementary Figure S1.

As shown in Supplementary Figure S1, the trend of change in the brain involvement index during the entire training process was generally consistent between the two groups, with the experimental group being slightly higher than the control group, especially after the second week of training, where a significant increase in the brain involvement index was observed. Statistical analysis of the changes in the brain involvement index throughout the training is presented in Table 7. From Table 7, it can be seen that the brain involvement index (16) in both groups was significantly higher than baseline after training (*p* < 0.05), with the experimental group showing a greater increase. Moreover, there were no statistical differences in the brain involvement index between the two groups before training, but after 4 weeks of training, a significant difference emerged, suggesting that during the training process, patients' brain involvement index showed varying degrees of increase.

**Table 7:** Changes in brain engagement index before and after training in both groups.

**Group**	**Session 1**	**Session 20**	***p*-value**
Control group	44.36 ± 12.43	74.80 ± 20.75	0.0458
Experimental group	43.15 ± 14.47	78.17 ± 18.07	0.0216
*p*-value	0.2130	0.0417	

**Figure 7:**
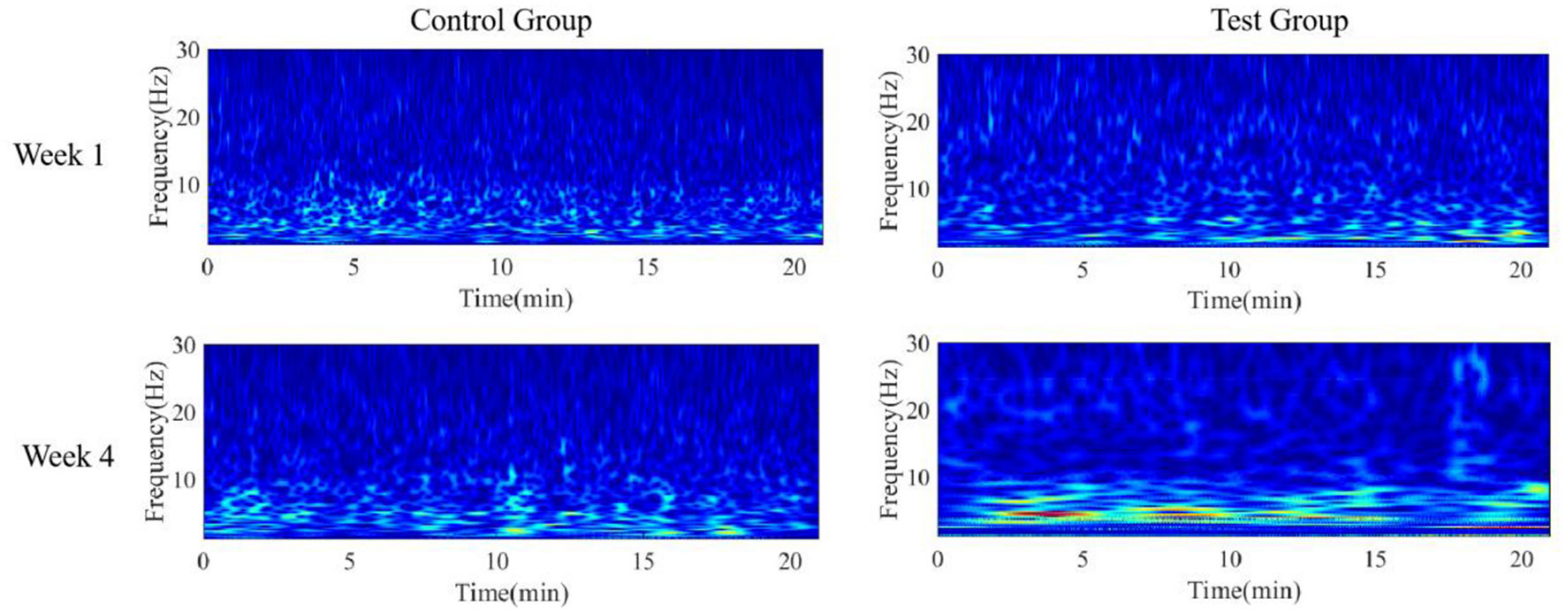
Time-frequency analysis of EEG signals from Cz channel. Time-frequency representations were derived from the Cz electrode, which corresponds to the motor cortex area associated with lower-limb movement. Given the motor imagery paradigm used in this study and the 4-week training duration, EEG data from the first session in week 1 and the last session in week 4 were analyzed to assess longitudinal changes.

SCI results in the loss of lower limb motor function in patients. Given that the Cz electrode corresponds to brain regions related to lower limb motor function (17) and the motor training paradigm in this study involves lower limb circular movements based on MI, we selected the EEG signal from the Cz electrode channel for time-frequency feature analysis. Considering the 4-week training period, the first training session in Week 1 and the last training session in Week 4 were chosen as evaluation points for time-frequency analysis of the Cz channel, with the results shown in Figure 7.

## 5 Discussion

The results of this study demonstrated that, after 4 weeks of treatment, the experimental group showed significantly greater improvements than the control group in multiple functional assessment indicators, including ASIA motor scores, ASIA sensory scores, BBS scores, and FAC walking function grades. These findings suggest that, compared to visual-induced MI training alone, BCI-based rehabilitation combined with conventional therapy more effectively promotes neurological recovery in patients with iSCI.

Mechanistically, BCI training enhances cortical functional reorganization and neuroplasticity by integrating real-time EEG signal acquisition with both visual and motor feedback. This approach facilitates more precise expression of motor intentions and more efficient transmission of descending signals. It not only improves connectivity within the cortex-spinal cord-muscle pathway but also potentially enhances activation of sensorimotor cortical regions, thereby promoting coordinated recovery of sensory and motor functions (18, 19).

Although the control group also exhibited partial improvements, indicating that MI combined with passive training can activate central nervous function to some extent, its effect appeared limited. This may be due to the absence of sufficient external feedback mechanisms, which hinders sustained cortical activation and the formation of an effective closed-loop regulatory system. In contrast, BCI systems provide continuous, perceivable positive feedback, enhancing the brain's awareness and modulation of “motor output outcomes,” thus accelerating the process of functional recovery (20).

Moreover, the BBS scores significantly improved in the experimental group but not in the control group, further indicating the positive impact of BCI training on the restoration of balance function. This may be related to its indirect facilitation of the cerebellar-vestibular pathways and proprioceptive feedback regulation (21). So, the BCI-mediated closed-loop neuromodulation strategy demonstrates superior potential in promoting functional recovery in patients with iSCI, particularly in the domains of motor control and gait performance.

Following SCI, the disruption of afferent neural input often leads to cortical denervation hypersensitivity, resulting in abnormal changes in cortical excitability (22). Theta rhythms, as a form of low-frequency EEG activity, are typically associated with cognitive processes, attentional regulation, and the encoding of sensory input. Their amplitude tends to decrease when cortical excitability is heightened. In contrast, high-frequency components such as beta waves are often enhanced under conditions of increased cortical activation and are closely related to motor planning, focused attention, and general arousal of the nervous system (23, 24). Therefore, after SCI, due to reduced somatosensory feedback and the initiation of compensatory mechanisms in the brain, a concurrent decrease in theta activity and increase in beta activity may occur. This pattern likely reflects the brain's initial adaptive response to neural injury.

The findings of this study indicate that, following training, both groups of patients exhibited increased relative power of theta rhythms across various cortical regions, suggesting a reduction in cortical excitability and a potential alleviation of neural hypersensitivity. Concurrently, beta activity showed a general downward trend, which may reflect a gradual decline in compensatory activity within motor planning-related areas as rehabilitation progressed. This trend implies that neural pathways within the injured regions may have undergone partial reconstruction, or that peripheral neural functions have been reorganized through alternative mechanisms. These EEG rhythm changes suggest that rehabilitation training may have contributed, at least in part, to the restoration of neural regulatory balance.

From an anatomical and functional perspective, the spinal cord, as an extension of the brain, constitutes an essential component of the central nervous system. It communicates bidirectionally with the brain through ascending and descending pathways. SCI not only causes direct loss of sensory and motor functions but also induces secondary changes in cortical structure and function, including neuronal apoptosis, synaptic dysfunction, and network remodeling (25). Moreover, the interaction between the brain and spinal cord is highly dynamic: SCI may weaken the brain's regulatory control over the body, while alterations in brain function may, in turn, influence spinal cord plasticity and recovery potential (26). Therefore, rehabilitation strategies should comprehensively address neural adaptation and reorganization at both the cerebral and spinal levels.

In this study's brain network analysis, the experimental group demonstrated beneficial changes in connectivity patterns within the theta and beta frequency bands following training. These changes were characterized by increased network density and enhanced functional reorganization, particularly in brain regions associated with motor intention encoding, sensory integration, and attentional modulation. These findings suggest that rehabilitation training promotes plastic reconfiguration of neural networks, facilitating the reintegration of damaged regions with residual pathways and thereby improving motor and sensory function. The enhancement of functional brain connectivity may serve as an important neurophysiological marker for evaluating training efficacy and provides electrophysiological evidence supporting the scientific rationale of the rehabilitation protocol (27).

Notably, compared with the control group, BCI training yielded more pronounced improvements in cortical activation patterns and network connectivity. By leveraging real-time feedback and EEG signal-driven control, the BCI system strengthened functional connectivity between the primary motor cortex and premotor areas, while also enhancing information integration between the sensory and motor cortices. This modulation increased the efficiency of motor control and sensory processing. Even in cases of partial disruption in neural conduction, BCI may compensate by activating residual pathways or facilitating the construction of new networks, thus demonstrating superior neuromodulatory potential beyond that of conventional training approaches.

From a neurophysiological standpoint, BCI-based rehabilitation may act on the nervous system through multiple mechanisms across various dimensions. First, long-term training can induce cortical plasticity, including the reorganization of neural circuits and optimization of activity patterns, thereby providing a structural foundation for functional recovery. Second, BCI training may promote synaptic remodeling, upregulate neurotrophic factor expression, and modulate neurotransmitter levels, thereby enhancing the efficiency of neural signal transmission and improving overall coherence and stability within the functional brain network. Ultimately, these neuromodulatory processes collectively drive adaptive cortical reorganization, offering potential improvements in both sensory and motor function for patients with iSCI (4, 28).

The advanges of EEG are as followed: High temporal resolution: EEG can directly record the electrical activities of neurons, with a temporal resolution reaching the millisecond level. It can capture the dynamic changes in the brain (such as rapid neural synchronization during cognitive tasks), which is difficult to achieve with functional magnetic resonance imaging (fMRI, with a resolution on the order of seconds) and functional near–infrared spectroscopy (fNIRS, with a resolution on the order of hundreds of milliseconds); Non–invasiveness and portability: EEG devices are small in size and relatively low in cost. They can be used in natural environments (such as daily activities and sleep monitoring), imposing few restrictions on the movements of the subjects. They are particularly suitable for children, the elderly, or long–term follow–up studies; Direct reflection of neural electrical activities: EEG signals originate from the summation of postsynaptic potentials of neurons, directly reflecting the electrophysiological mechanisms of neural activities. In contrast, fMRI and fNIRS infer brain functions through hemodynamic responses (indirect indicators) (29, 30).

In summary, this study demonstrates that BCI rehabilitation training based on a visually guided MI paradigm not only improves EEG activity patterns and cortical excitability in patients with iSCI, but also promotes orderly neural network reconstruction through enhanced functional connectivity and activation of specific motor-related cortical regions. BCI training exhibits significant neuromodulatory capabilities and represents a promising neurorehabilitation strategy worthy of further investigation and broader clinical application.

## 6 Limitations and future directions

One of the primary limitations of this study lies in the relatively small sample size, as it was conducted as a single-center trial with a limited number of participants. This may affect the generalizability of the findings and reduce the statistical power of the results. This study is a small-sample exploratory trial with limited statistical power. The results should be regarded as preliminary evidence and need to be verified by future large-sample studies. Nevertheless, as an exploratory investigation, this study provides valuable preliminary data and conceptual groundwork for future large-scale, multicenter randomized controlled trials. In addition, the present study primarily employed EEG-based indices such as brain engagement metrics and time-frequency analyses to evaluate the effects of BCI intervention. Future research should consider incorporating multimodal neurophysiological assessments-including functional near-infrared spectroscopy, functional magnetic resonance imaging, and electromyography (EMG)-to more comprehensively elucidate the mechanisms through which BCI training modulates the central nervous system. Then, the non-neuronal factors such as volume conduction and common input on phase-dependent relationships are crucial considerations in the interpretation of EEG signals. Finally, although the current study evaluated the effects of a 4-week intervention, it did not include long-term follow-up, making it difficult to assess the sustained efficacy of BCI training or its role in long-term functional maintenance. Future studies should extend the follow-up period to investigate the durability of BCI-induced improvements in functional recovery and neural reorganization over time. Additionally, this study did not delve deeply into the differences and mechanisms of neural plasticity among different spinal cord segments (cervical, thoracic, and lumbar). However, we believe that the inherent differences in the molecular phenotypes (such as the expression of ion channels and neuropeptides) of DRG neurons in different spinal cord segments may lead to heterogeneous responses after injury. The descending regulatory intensities from the brainstem and cerebral cortex on the upper spinal cord (such as the cervical segment) and the lower spinal cord (such as the lumbar segment) are different, which may affect the expression of plasticity-related genes. The differences in the activation patterns of glial cells, the release profiles of inflammatory factors, and blood supply in different spinal cord segments may further amplify the segmental specificity of neural plasticity. Future studies can analyze the regulatory mechanisms of spinal cord segments on neural plasticity through cross-segmental comparative designs, providing a theoretical basis for precise targeted therapy. We should also pay attention to that the comparison between non-invasive MI-BCI and invasive brain-spine interfaces could provide a critical contextualization of the current research within the broader field of neurorehabilitation.

## 7 Conclusion

This study demonstrated the feasibility and efficacy of visually guided MI–based BCI rehabilitation training in patients with iSCI. The results showed that BCI training significantly enhanced brain engagement and promoted functional reorganization in cortical regions associated with motor control and sensory integration. Participants in the experimental group outperformed those in the control group across multiple neurophysiological indicators and clinical functional assessments. Further analysis revealed that BCI training may engage neuroplastic mechanisms by enhancing functional brain network connectivity, reducing cortical excitability, and optimizing EEG rhythms, thereby facilitating compensatory reorganization of neural pathways. These findings provide preliminary evidence for understanding the mechanisms by which BCI contributes to neurological functional recovery. No adverse events were observed during the study, indicating that this rehabilitation intervention is safe and well-tolerated in clinical settings. Future studies should be conducted with larger sample sizes and across multiple centers to further elucidate the mechanistic effects of BCI in functional restoration following iSCI, and to evaluate its clinical applicability and potential for broader implementation in rehabilitation practice.

## Data Availability

The original contributions presented in the study are included in the article/Supplementary material, further inquiries can be directed to the corresponding author.

## Supplementary material

Supplementary Figure S1 Changes in brain engagement index during training. The brain engagement index (BEI), reflecting the degree of cortical activation during motor imagery, was continuously monitored across 20 training sessions (five sessions per week for 4 weeks). In the experimental group, the BEI was used to drive the lower-limb rehabilitation robot in real time, enabling cyclic leg movements based on participants' brain activity, thus providing continuous neurofeedback through movement speed. In contrast, the control group did not receive such real-time movement feedback, as the robot operated at a fixed passive speed irrespective of brain activity.

## References

[1] Sterner RC Sterner RM. Immune response following traumatic spinal cord injury: pathophysiology and therapies. Front Immunol. (2022) 13:1084101. doi: 10.3389/fimmu.2022.1084101 36685598 PMC9853461

[2] Li F Wei C Huo S Liu X Du J. Noninvasive brain stimulation for motor dysfunction after incomplete spinal cord injury: a systematic review and meta-analysis. Am J Phys Med Rehabil. (2024) 103:53–61. doi: 10.1097/PHM.0000000000002311 37408131

[3] Cajigas I Vedantam A. Brain-computer interface, neuromodulation, and neurorehabilitation strategies for spinal cord injury. Neurosurg Clin N Am. (2021) 32:407–17. doi: 10.1016/j.nec.2021.03.012 34053728

[4] Kazim SF Bowers CA Cole CD Varela S Karimov Z Martinez E . Corticospinal motor circuit plasticity after spinal cord injury: harnessing neuroplasticity to improve functional outcomes. Mol Neurobiol. (2021) 58:5494–516. doi: 10.1007/s12035-021-02484-w 34341881

[5] Bao G Pan L Fang H Wu X Yu H Cai S . Academic review and perspectives on robotic exoskeletons. IEEE Trans Neural Syst Rehabil Eng. (2019) 27:2294–304. doi: 10.1109/TNSRE.2019.2944655 31567097

[6] Romero-Laiseca MA Delisle-Rodriguez D Cardoso V Gurve D Loterio F Posses Nascimento JH . A low-cost lower-limb brain-machine interface triggered by pedaling motor imagery for post-stroke patients rehabilitation. IEEE Trans Neural Syst Rehabil Eng. (2020) 28:988–96. doi: 10.1109/TNSRE.2020.2974056 32078552

[7] Colucci A Vermehren M Cavallo A Angerhofer C Peekhaus N Zollo L . Brain-computer interface-controlled exoskeletons in clinical neurorehabilitation: ready or not? Neurorehabil Neural Repair. (2022) 36:747–56. doi: 10.1177/15459683221138751 36426541 PMC9720703

[8] Blanco-Diaz CF Serafini E Bastos-Filho T Dantas A Santo C Delisle-Rodriguez D . Gait imagery-based brain-computer interface with visual feedback for spinal cord injury rehabilitation on lokomat. IEEE Trans Biomed Eng. (2025) 72:102–11. doi: 10.1109/TBME.2024.3440036 39110553

[9] Cui Z Li Y Huang S Wu X Fu X Liu F . BCI system with lower-limb robot improves rehabilitation in spinal cord injury patients through short-term training: a pilot study. Cogn Neurodyn. (2022) 16:1283–301. doi: 10.1007/s11571-022-09801-6 36408074 PMC9666612

[10] Leng J Yu X Wang C Zhao J Zhu J Chen X . Functional connectivity of EEG motor rhythms after spinal cord injury. Cogn Neurodyn. (2024) 18:3015–29. doi: 10.1007/s11571-024-10136-7 39555294 PMC11564577

[11] Holler Y Thomschewski A Uhl A Bathke AC Nardone R Leis S . HD-EEG based classification of motor-imagery related activity in patients with spinal cord injury. Front Neurol. (2018) 9:955. doi: 10.3389/fneur.2018.00955 30510537 PMC6252382

[12] Yuan Z Peng Y Wang L Song S Chen S Yang L . Effect of BCI-controlled pedaling training system with multiple modalities of feedback on motor and cognitive function rehabilitation of early subacute stroke patients. IEEE Trans Neural Syst Rehabil Eng. (2021) 29:2569–77. doi: 10.1109/TNSRE.2021.3132944 34871175

[13] Costa A Ianez E Ubeda A Hortal E Del-Ama AJ Gil-Agudo A . Decoding the attentional demands of gait through EEG gamma band features. PLoS ONE. (2016) 11:e0154136. doi: 10.1371/journal.pone.0154136 27115740 PMC4846000

[14] Decety J. The neurophysiological basis of motor imagery. Behav Brain Res. (1996) 77:45–52. doi: 10.1016/0166-4328(95)00225-1 8762158

[15] Simis M Uygur-Kucukseymen E Pacheco-Barrios K Battistella LR Fregni F. Beta-band oscillations as a biomarker of gait recovery in spinal cord injury patients: a quantitative electroencephalography analysis. Clin Neurophysiol. (2020) 131:1806–14. doi: 10.1016/j.clinph.2020.04.166 32540720

[16] Wan C Zhang Q Qiu Y Zhang W Nie Y Zeng S . Effects of dual-task mode brain-computer interface based on motor imagery and virtual reality on balance and attention in patients with stroke: a randomized controlled pilot trial. J Neuroeng Rehabil. (2025) 22:187. doi: 10.1186/s12984-025-01730-9 40883792 PMC12395916

[17] Arunganesh K Sivakumaran N Kumaravel S Karthick P. Analysis of EEG-EMG coherence in low frequency bands. Stud Health Technol Inform. (2021) 281:520–1. doi: 10.3233/SHTI210224 34042630

[18] Mohammed H Hollis ER 2nd. Cortical reorganization of sensorimotor systems and the role of intracortical circuits after spinal cord injury. Neurotherapeutics. (2018) 15:588–603. doi: 10.1007/s13311-018-0638-z 29882081 PMC6095783

[19] Urbin MA Royston DA Weber DJ Boninger ML Collinger JL. What is the functional relevance of reorganization in primary motor cortex after spinal cord injury? Neurobiol Dis. (2019) 121:286–95. doi: 10.1016/j.nbd.2018.09.009 30217521

[20] Shupe LE Miles FP Jones G Yun R Mishler J Rembado I . Neurochip3: an autonomous multichannel bidirectional brain-computer interface for closed-loop activity-dependent stimulation. Front Neurosci. (2021) 15:718465. doi: 10.3389/fnins.2021.718465 34489634 PMC8417105

[21] Cabaraux P Agrawal SK Cai H Calabro RS Casali C Damm L . Consensus paper: ataxic gait. Cerebellum. (2023) 22:394–430. doi: 10.1007/s12311-022-01373-9 35414041

[22] Moxon KA Oliviero A Aguilar J Foffani G. Cortical reorganization after spinal cord injury: always for good? Neuroscience. (2014) 283:78–94. doi: 10.1016/j.neuroscience.2014.06.056 24997269 PMC4556279

[23] Clarke AR Barry RJ Karamacoska D Johnstone SJ. The EEG theta/beta ratio: a marker of arousal or cognitive processing capacity? Appl Psychophysiol Biofeedback. (2019) 44:123–9. doi: 10.1007/s10484-018-09428-6 30604100

[24] Pefkou M Arnal LH Fontolan L Giraud AL. Theta-band and beta-band neural activity reflects independent syllable tracking and comprehension of time-compressed speech. J Neurosci. (2017) 37:7930–8. doi: 10.1523/JNEUROSCI.2882-16.2017 28729443 PMC6596908

[25] Calderone A Cardile D De Luca R Quartarone A Corallo F Calabro RS. Brain plasticity in patients with spinal cord injuries: a systematic review. Int J Mol Sci. (2024) 25:2224. doi: 10.3390/ijms25042224 38396902 PMC10888628

[26] Lemon RN. Descending pathways in motor control. Annu Rev Neurosci. (2008) 31:195–218. doi: 10.1146/annurev.neuro.31.060407.125547 18558853

[27] Zhan G Chen S Ji Y Xu Y Song Z Wang J . EEG-based brain network analysis of chronic stroke patients after BCI rehabilitation training. Front Hum Neurosci. (2022) 16:909610. doi: 10.3389/fnhum.2022.909610 35832876 PMC9271662

[28] Krucoff MO Rahimpour S Slutzky MW Edgerton VR Turner DA. Enhancing nervous system recovery through neurobiologics, neural interface training, and neurorehabilitation. Front Neurosci. (2016) 10:584. doi: 10.3389/fnins.2016.00584 28082858 PMC5186786

[29] Wang X Li W Song R Ao D Hu H Li L. Corticomuscular coupling alterations during elbow isometric contraction correlated with clinical scores: an fNIRS-sEMG study in stroke survivors. IEEE Trans Neural Syst Rehabil Eng. (2025) 33:696–704. doi: 10.1109/TNSRE.2025.3535928 40031336

[30] Li L Wang X Yao B Zhang X Zhou P. Sample entropy-based surface electromyographic examination with a linear electrode array in survivors with spinal cord injury. IEEE Trans Neural Syst Rehabil Eng. (2023) 31:2944–52. doi: 10.1109/TNSRE.2023.3290607 37379182

