# Supplementary figures and images for: Effects of visually induced motor imagery-based brain-computer interface training on motor function in patients with incomplete spinal cord injury: a small-sample exploratory trial
Source: Front Neurol. 2026 Feb 3;17:1700249. doi: 10.3389/fneur.2026.1700249 (PMC12909217; doi:10.3389/fneur.2026.1700249)

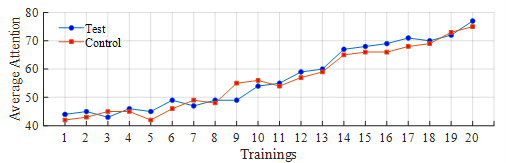

Supplement: Supplementary Figure S1 — Changes in brain engagement index during training. The brain engagement index (BEI), reflecting the degree of cortical activation during motor imagery, was continuously monitored across 20 training sessions (five sessions per week for 4 weeks). In the experimental group, the BEI was used to drive the lower-limb rehabilitation robot in real time, enabling cyclic leg movements based on participants' brain activity, thus providing continuous neurofeedback through movement speed. In contrast, the control group did not receive such real-time movement feedback, as the robot operated at a fixed passive speed irrespective of brain activity. [file Image_1.tif]
